# STAT3 and NF-κB cooperatively control *in vitro* spontaneous apoptosis and poor chemo-responsiveness in patients with chronic lymphocytic leukemia

**DOI:** 10.18632/oncotarget.8672

**Published:** 2016-04-09

**Authors:** Feng-Ting Liu, Li Jia, Ping Wang, Huaqing Wang, Timothy W. Farren, Samir G. Agrawal

**Affiliations:** ^1^ Department of Radiobiology, Key Laboratory of Cancer Prevention and Therapy, National Clinical Research Centre for Cancer, Tianjin Medical University Cancer Institute and Hospital, Tianjin, China; ^2^ Centre for Haemato-Oncology, Barts Cancer Institute, Queen Mary University of London, London, UK; ^3^ Department of Medical Oncology, Tianjin Union Medicine Center, Tianjin, China; ^4^ Pathology Group, Blizard Institute, Queen Mary University of London, London, UK

**Keywords:** spontaneous apoptosis, Chronic lymphocytic leukemia, STAT3, NF-kB, prognosis

## Abstract

Chronic lymphocytic leukemia (CLL) is an adult disease characterized by *in vivo* accumulation of mature CD5/CD19/CD23 triple positive B cells and is currently incurable. CLL cells undergo spontaneous apoptosis in response to *in vitro* cell culture condition but the underlying mechanism is unclear. We hypothesize that the sensitivity of CLL cells to spontaneous apoptosis may be associated with the constitutive activities of transcription factors STAT3 and/or NF-κB. We now show that the sensitivity of fresh CLL cells to spontaneous apoptosis is highly variable among different patients during 48 hours’ cell culture and inversely correlated with *in vivo* constitutively activated STAT3 and NF-κB (*p* < 0.001). Both activated STAT3 and NF-κB maintain the levels of anti-apoptotic protein Mcl-1/Bcl-xL and autocrine IL-6 production. CLL cells with higher susceptibility to *in vitro* spontaneous apoptosis show the greatest chemosensitivity (*p* < 0.001), which is reflected clinically as achieving a complete response (CR) (*p* < 0.001), longer lymphocyte doubling times (*p* < 0.01), time to first treatment (*p* < 0.01), and progression free survival (*p* < 0.05). Our data suggest that the sensitivity of CLL cells to *in vitro* spontaneous apoptosis is co-regulated by constitutively activated STAT3 and NF-κB and reflects the *in vivo* chemo-responsiveness and clinical outcomes.

## INTRODUCTION

Chronic lymphocytic leukemia (CLL) is the most common form of adult leukemia in western countries, and is characterized by the progressive accumulation of phenotypically mature monoclonal CD5+ malignant B-lymphocytes in peripheral blood (PB), bone marrow (BM), and lymphoid organs [1, 2]. Although there are improvements in clinical outcomes with current chemo-immunotherapy, CLL remains incurable. Understanding the mechanisms that contribute to the survival and chemoresistance of CLL cells could lead to new and more effective therapeutic strategies.

Despite their longevity *in vivo*, circulating CLL cells rapidly undergo spontaneous apoptosis *in vitro* [3-6], indicating that *in vitro* conditions lack essential survival factors which are present *in vivo*. Many factors have been reported to reduce *in vitro* spontaneous apoptosis: such as bone marrow stromal cells [7, 8], nurse-like cells [9, 10], T cells [11] and dendritic cells [12] *via* cell-cell contact or secreted cytokines. The intracellular aberrant expression of Bcl-2 family of proteins, such as Bcl-2 [13], Mcl-1 [8] or Bcl-xL [4, 14-16] are associated with poor chemo-responsiveness and clinical prognosis as well as a decreased *in vitro* survival.

Transcription factors STAT3 and NF-κB are aberrantly activated in many cancer cells. These tumor-associated transcription factors co-regulate gene expression, resulting in significant upregulation of genes involved in tumor cell survival, proliferation, and immunosuppression [17, 18]. NF-κB family proteins include RelA (p65), RelB, c-Rel, p50 (NF-κB1), and p52 (NF-κB2) transcription factors [17, 19]. The most commonly detected NF-κB dimer is RelA/p50 that is responsible for the processed a strong transcriptional activation domain and RelA is responsible for most of NF-κB transcriptional activity [19]. The constitutive phosphorylation of STAT3 on tyrosine 705 residues (p-STAT3^Y705^) is found in a wide variety of human cancer cells [20, 21]. However, in fresh primary CLL cells, p-STAT3^Y705^ is not detectable, whereas phosphorylation of STAT3 on serine 727 residues (p-STAT3^S727^) is constitutively expressed in all CLL cases [22, 23]. The mechanisms underlying constitutive p-STAT3^S727^ expression in CLL cells remain unclear. Studies have shown that IL-6 induces STAT3 phosphorylation on both Tyr705 and Ser727 [23, 24]. The nuclear p-STAT3^S727^ binds to DNA and mediates gene transcription [23], while mitochondrial p-STAT3^S727^ regulates mitochondrial respiration [25, 26].

Both STAT3 and NF-κB regulate the production of many cytokines, including IL-6, IL-8, IL-17, IL-21 and IL-23, and expression of anti-apoptotic proteins [27-29]. Many types of cancer cells can secrete IL-6 because of the constitutively activated STAT3 and NF-κB [30]. Autocrine and paracrine of IL-6 in turn maintain STAT3 and NF-κB activities through direct or indirect signal pathways [31-33]. Although the cross-talking between STAT3 and NF-κB can further promote cancer cell proliferation and survival [17, 18, 33], it is unknown whether co-regulation of STAT3 and NF-κB plays an important role in CLL cell survival and disease progression.

Here, we aimed to determine whether constitutive activation of both STAT3 and NF-κB co-regulates *in vitro* CLL cell survival and disease progress in patients with CLL. We report for the first time that the sensitivity of CLL cells to spontaneous apoptosis reflects chemo-responsiveness and disease progression in patients with CLL.

## RESULTS

### Differential sensitivities of CLL cells to *in vitro* spontaneous apoptosis

**Figure 1:**
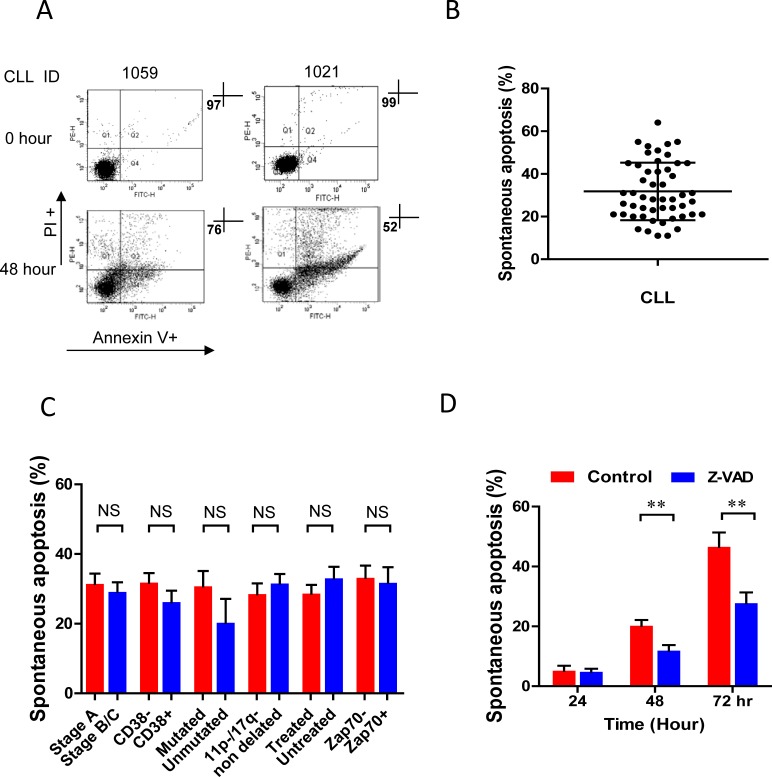
*In vitro* spontaneous apoptosis of fresh CLL cells Freshly isolated CLL cells were incubated in complete culture medium for 48 hours. **A.** Apoptotic cell death was measured by flow cytometry at 0 hour (without incubation) and after *in vitro* incubation for 48 hours in 2 CLL cases. **B.** Apoptotic cell death in 51 cases was determined by flow cytometry (mean ± SD). **C.** The level of spontaneous apoptosis in different clinical prognostic biomarker groups: Binet stage at presentation (A *vs* B/C, *n* = 51), treated *vs* untreated (*n* = 51), CD38 (negative or positive; cut-off 20%, *n* = 47), cytogenetic (absence or presence of 11q and 17p deletions, *n* = 48), ZAP-70 (negative or positive; cut-off 20%, *n* = 26) and IgHV mutated *vs* unmutated (*n* = 16) cases. **D.** CLL cells were incubated with or without 20μM Z-VAD.fmk for 24, 48 and 72 hours, then spontaneous apoptosis was analyzed by flow cytometry (*n* = 6). ** *p* < 0.01, ***p < 0.001.

CLL is a disease of *in vivo* accumulation and CLL cells undergo rapid spontaneous apoptosis in the cultured condition [3, 6, 34]. We aimed to determine whether the sensitivity to spontaneous apoptosis varies among different CLL cases. The spontaneous apoptosis was tested *in vivo* (0 hour) and *in vitro* under cultured condition on 51 fresh CLL samples. The sensitivities of CLL cells to spontaneous apoptosis were variable in individual CLL cases (Figure 1A), - the average spontaneous apoptosis over 51 CLL cases was 31.8%±13.5 at 48 hours’ time point (Figure 1B). The sensitivity of CLL cells to spontaneous cell death is not significantly correlated with Binet stage, treatment status, IgHV mutation, cytogenetic analysis, and the positivity of CD38 and ZAP70 (Figure 1C). Treatment with pan caspase inhibitor Z-VAD.fmk significantly decreased percentages of CLL cells that underwent spontaneous apoptosis (Figure 1D). While the data suggest that the intercellular survival signals play important role for spontaneous apoptosis in CLL cell survival, there was marked variation between different cases, implying that the degree of *in vitro* spontaneous apoptosis may reflect the underlying biology of the disease, as well as loss of *in vivo* signals.

### CLL cells produce IL-6 during *in vitro* culture

**Figure 2:**
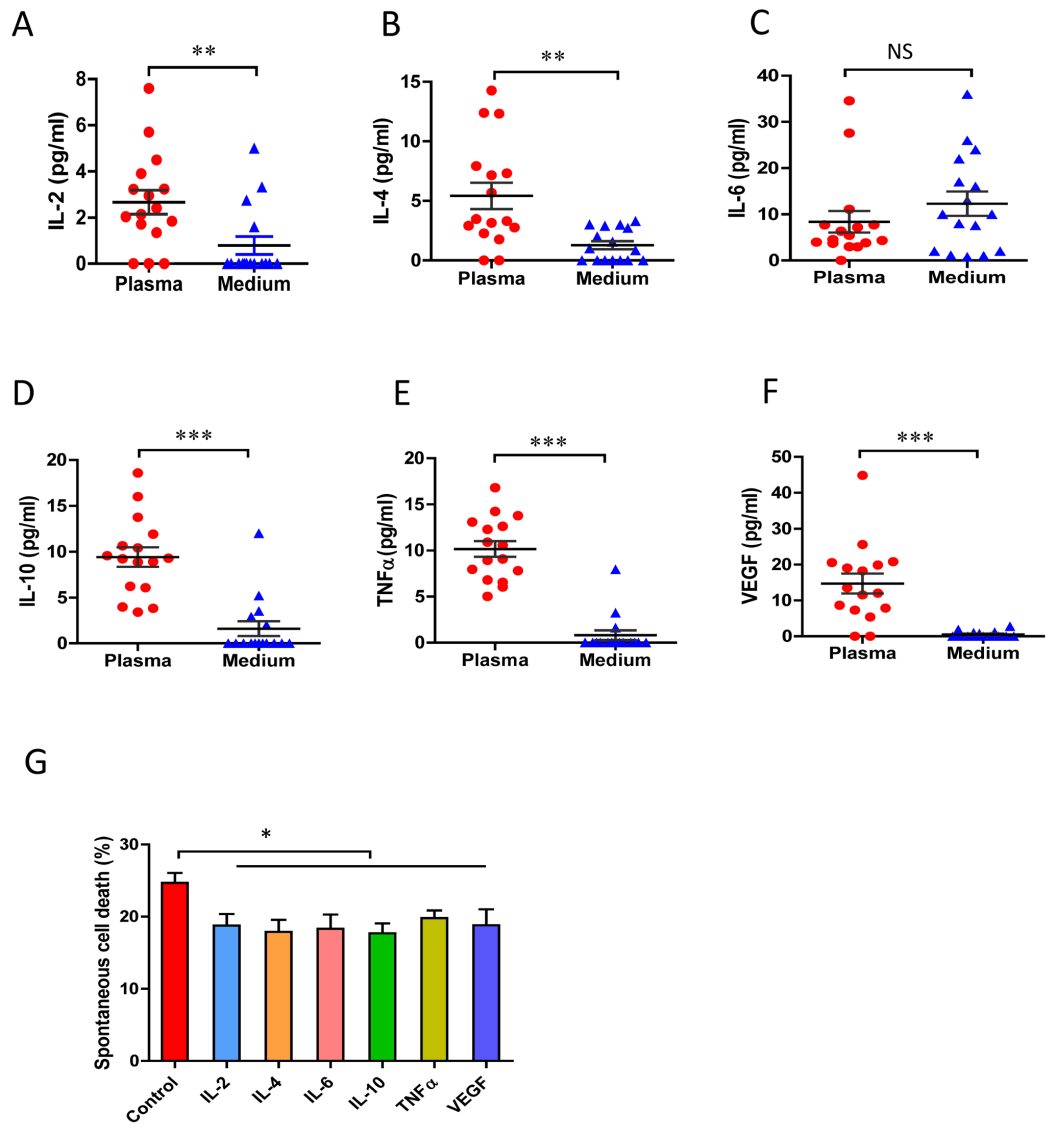
*In vivo* and *in vitro* cytokine production by CLL cells 5×10^5^/ml of fresh CLL cells (CD19+/CD5+ more than 95%) were cultured in RPMI-1640 complete medium for 24 hours. The conditioned medium and corresponding plasma samples were incubated with cytokine beads and the levels of cytokine production were measured by flow cytometry: **A.** IL-2, **B.** IL-4, **C.** IL-6, **D.** IL-10, **E.** TNF-α and **F.** VGEF concentrations (*n* = 16). **G.** CLL cells were incubated with 10ng/ml of IL-2, 10ng/ml of IL-4, 10ng/ml of IL-6, 10ng/ml of IL-10, 10ng/ml of TNF-α or 10ng/ml of VGEF for 48 hours. Decreased percentages of apoptotic cell death mediated by cytokines were assessed by flow cytometry and compared with the untreated controls (*n* = 5). * *p* < 0.05, ** *p* < 0.01, ***p < 0.001.

CLL cells are long-lived in the circulation with survival signals from the protective microenvironment [3, 4]. Previous studies demonstrated the prevention of CLL cells from apoptosis is associated with presence of cytokines, such as IL-2, IL-4 [34], IL-6 [35], IL-10 [36], VEGF [37] and/or TNFα [38]. The levels of cytokine production, including IL-2, IL-4, IL-6, IL-10, TNFα and VEGF were determined in the plasma of patients and the supernatants from the cell culture. The levels of all cytokines in the cultured medium were significantly lower than those in the plasma, with many being undetectable in most cases (Figure 2A-2F), with the only exception of IL-6, which was similar in the supernatant and in the plasma (Figure 2C). All these cytokines have been reported to regulate *in vivo* cell growth and survival [34-36]. After *in vitro* treatment with IL-2, IL-4, IL-6, IL-10, TNFα or VEGF individually for 48 hours, all those cytokines significantly reduced the extents of CLL cell's to spontaneous apoptosis (Figure 2G). Our previous study found that engagement of CD160 greatly increased IL-6 production but no other cytokine [4]. These data indicate that *in vitro* spontaneous apoptosis of CLL may be related to the loss of multiple microenvironmental signals, while autocrine IL-6 might play an important role for *in vitro* survival of CLL cells.

### Autocrine IL-6 production is co-regulated by STAT3 and NF-κB activities in CLL cells

**Figure 3:**
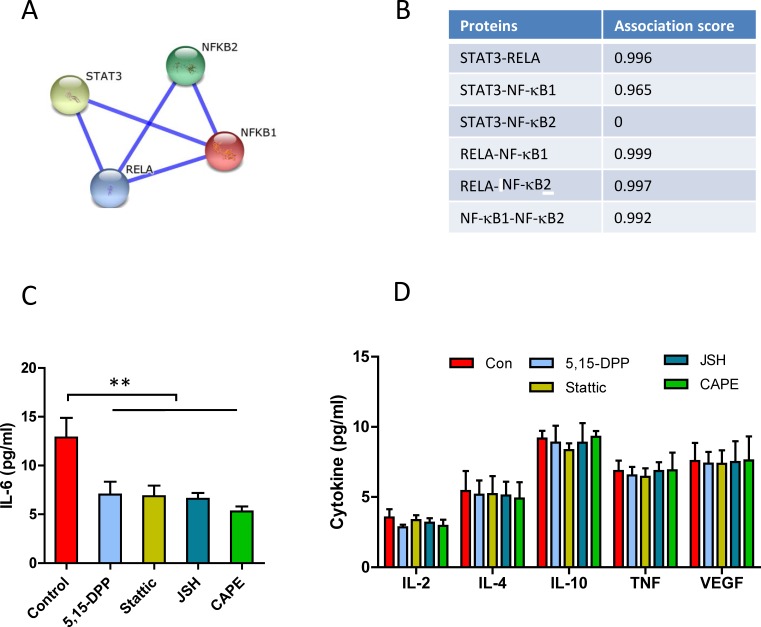
Co-regulation of STAT3 and NF-κB on autocrine IL-6 production **A.** and **B.** Prediction of the interaction between STAT3 and NF-κB by STRING v10 database. **A.** Interaction network of STAT3 and NF-κB. The interaction network is generated by manual input of multiple human transcriptional factors. The thickness of the connection line reflects the binding score of two proteins. **B.** Combined association scores, including physical and functional interaction. **C. D.** Fresh CLL cells were incubated with 10μM of Stattic, 10μM of 5,15-DDP, 10μM of CAPE or 10μM of JSH-23 for 24 hours in complete PRMI-1640 medium respectively. **C.** Autocrine IL-6 production (*n* = 9); **D.** IL-2, IL-4, IL-10, TNFα or VEGF (*n* = 4) production in the conditioned medium was determined by human CBA Flex kit and analyzed by flow cytometry.

STAT3 and NF-κB co-regulate IL-6 production in different cell types [30]. The interactions between STAT3 and NF-κB family of proteins were predicted by the STRING v10 database (http://string-db.org/) [39]. STAT3 is highly interactive (both physically and functionally) with both RelA and NF-κB1 but not NF-κB2 (Figure 3A and 3B). About 123 genes are co-regulated by both NF-κB and STAT3 [32]. The promoter region of IL-6 gene contains binding sites of STAT3, RelA and NF-κB1 (http://www.sabiosciences.com/). To explore whether the autocrine IL-6 were mediated by STAT3 and NF-κB activation in CLL, we tested the function of constitutively activated STAT3 and NF-κB by inhibiting transcriptional activities of STAT3 and NF-κB. Fresh CLL cells were treated with STAT3 inhibitors Stattic or 5,15-DPP and NF-κB inhibitors CAPE or JSH-23, respectively. The inhibition on either STAT3 transcription activity (by Stattic and 5,15-DPP) [40, 41] or RelA (by CAPE or JSH-23) [42, 43] significantly decreased autocrine IL-6 production (Figure 3C). The concentrations of inhibitors used for down-regulation of STAT3 and NF-κB did not significantly affect the autocrine of IL-2, IL-4, IL-10, TNFα and VGEF production (Figure 3D). These results indicate that autocrine IL-6 production was mediated by both STAT3 and NF-κB activation in CLL cells.

### STAT3 and RelA prevent CLL cell apoptosis *via* an increase in expression of Mcl-1 and Bcl-xL

**Figure 4:**
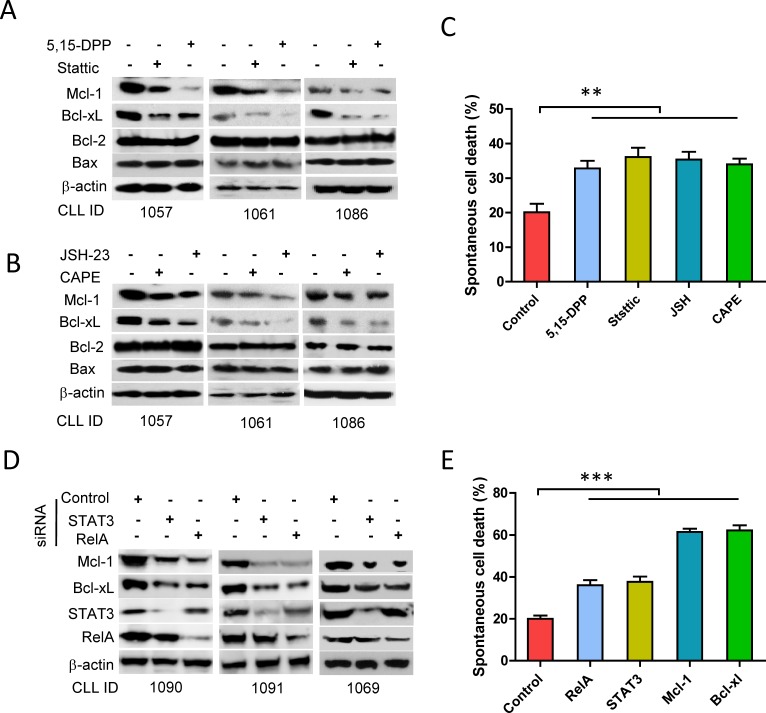
Regulation of STAT3 and RelA on Bcl-xL/Mcl-1 expression and spontaneous apoptosis Fresh CLL cells were incubated with 10μM of Stattic, 10μM of 5,15-DDP, 10μM of CAPE or 10μM of JSH-23 for 24 hours respectively. (A and B) Expression of Bcl-xL, Mcl-1, Bcl-2, and Bax was determined by Western blotting. **C.** Spontaneous apoptotic cell death for 48 hours incubation was detected by flow cytometry (*n* = 5). **D.** Down-regulation of STAT3 and RelA by siRNA. Cells were transfected with STAT3-siRNA, RelA-siRNA or control siRNA. Expression of Mcl-1, Bcl-xL, STAT3, and RelA were determined by Western blotting. **E.** Cells were transfected with STAT3-siRNA, RelA-siRNA, Mcl-1-siRNA, Bcl-xL-siRNA or control siRNA and spontaneous apoptosis was determined by flow cytometry after transfection for 24 hours(*n* = 5).

The anti-apoptotic proteins Mcl-1 and Bcl-xL are target genes for both STAT3 and NF-κB [30, 33, 44, 45]. Inhibition of either STAT3 by Stattic and 5,15-DDP or NF-κB by JSH-23 and CAPE showed similar inhibitory effect on the expression of Bcl-2 family proteins: i.e. decreased expression of Mcl-1 and Bcl-xL, but not Bcl-2 or Bax (Figure 4A and 4B). Global NF-κB and STAT3 activities were analyzed as the sum of p-RelA+p-STAT3 (Supplementary Figure 1A right panel). The levels of anti-apoptotic and pro-apoptotic proteins were analyzed accordingly in the same case (Supplementary Figure 1B). The CLL cases with greater p-STAT3+p-RelA expression also showed higher levels of Bcl-xL+Mcl-1 (Supplementary Figure 1B right panel). The levels of Mcl-1 plus Bcl-xL expression were significantly correlated with p-STAT3+p-RelA (*p* < 0.01, Supplementary Figure 1C). Although the inhibition of STAT3 and NF-κB did not alter Bcl-2 or Bax expression, the global impact of decreased Mcl-1 and Bcl-xL could directly result in an increased spontaneous apoptosis (Figure 4C). These results were confirmed by transient knockdown of STAT3 or NF-κB, which led to decreased expression of Mcl-1 and Bcl-xL (Figure 4D), and increased spontaneous apoptosis (Figure 4E). Transient knockdown of Mcl-1 and Bcl-xL for 24 hours results in a dramatic increase in spontaneous apoptosis in CLL cells (Figure 4E). Overall, these results demonstrate key roles of autocrine IL-6, expression of Mcl-1 and Bcl-xL - which are under the direct control of STAT3 and NF-κB activation in protecting against spontaneous apoptosis in CLL.

### The degrees of autocrine IL-6 and spontaneous apoptosis reflect the level of constitutive activities of STAT3 and NF-κB in CLL cells

**Figure 5:**
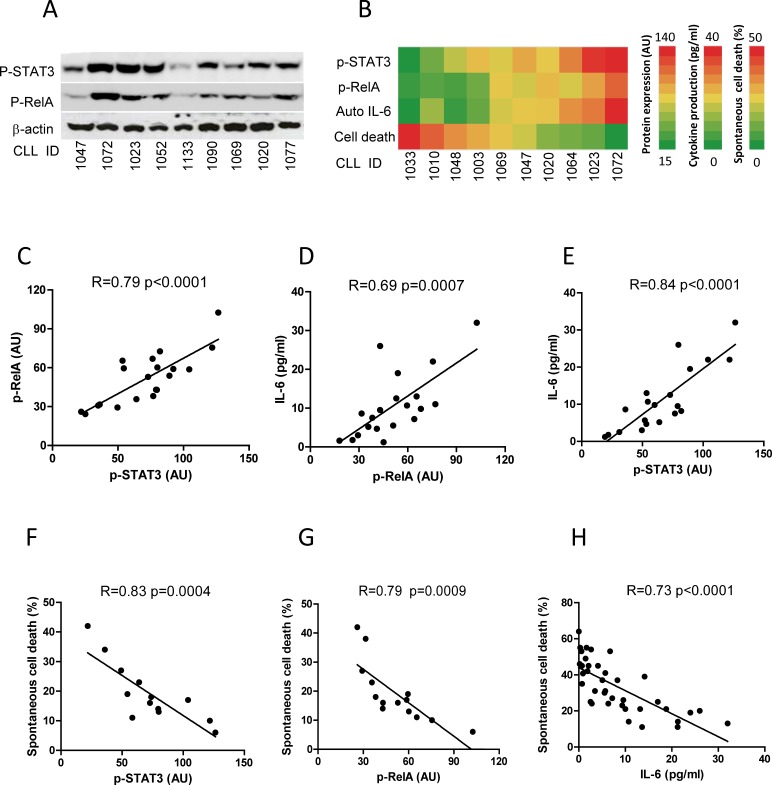
Correlation of constitutive activities of STAT3 and RelA with *in vitro* IL-6 production and spontaneous apoptosis **A.** Constitutive activities of STAT3 and RelA. Expression of p-STAT3 and p-RelA was determined on 9 fresh CLL samples by Western Blotting. **B.** The association of expression of p-STAT3/p-RelA with IL-6 production and spontaneous cell death from 10 CLL cases (CLL ID) was expressed by a heat map. The levels of p-STAT3 and p-RelA expression ranged from 15-140 arbitrary units (AU) (analyzed by densitometry using GelScan v 5.1 software); autocrine IL-6 ranged from 0-40pg/ml and cell death ranged from 0-50%. (C, D, E) Correlation between p-STAT3 and p-RelA **C.** the correlation of IL-6 production to p-RelA **D.** or p-STAT3 **E.** was analyzed in 20 CLL cases. Correlation between spontaneous cell death and p-STAT3 **F.** or p-RelA **G.** was analyzed in 14 CLL cases. **H.** Correlation between IL-6 production and spontaneous cell death was analyzed in 38 CLL cases.

In CLL, STAT3 is uniquely phosphorylated at serine-727 (p-STAT3^S727^) rather than at tyrosine 705 (p-STAT3^Y705^) [23]. Differential expression of p-STAT3^S727^ and p-RelA was determined by Western Blotting (Figure 5A). The heat map demonstrates that the levels of p-STAT3/p-RelA and autocrine IL-6 showed positive association, while the sensitivity of these cells to spontaneous cell death displayed a negative association with those three variables (Figure 5B). The correlation among these four variables of 20 cases of fresh CLL samples was statistically analyzed by the Pearson's correlation. The levels of p-STAT3, p-RelA and autocrine IL-6 were significantly correlated with each other (Figure 5C-5E), but higher expression of p-STAT3 or p-RelA was negatively and significantly correlated with the sensitivity of these CLL samples to spontaneous apoptosis (Figure 5F and 5G). This suggests that the corollary *in vitro* autocrine IL-6 and spontaneous apoptosis could be surrogate markers for *in vivo* STAT3 and NF-κB activities in CLL cells. To further explore the role of autocrine IL-6 in CLL cell survival, the IL-6 production and spontaneous apoptosis were determined in 38 CLL cases. The median production of IL-6 was 6.68 pg/ml (range, 0.2-32 pg/ml, Supplementary Figure 2A), and the median of spontaneous cell death was 31% (range, 11-64%, Supplementary Figure 2B). In general, CLL cells with higher IL-6 production (more than median) significantly correlated with less sensitivity to spontaneous cell death (Figure 5H). These results indicate that autocrine IL-6 not only reflects the degree of STAT3 and NF-κB activation, but also *in vitro* CLL cell survival.

### Constitutive activities of STAT3 and NF-κB negatively correlate with *in vitro* chemosensitivity of CLL cells

**Figure 6:**
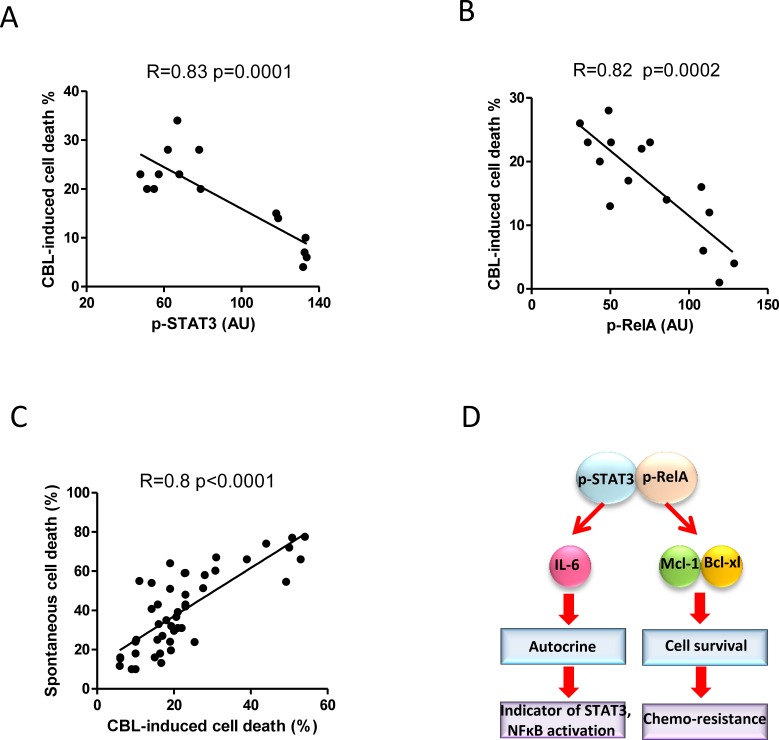
Correlation between constitutively activated STAT3 and RelA with chemosensitivity 5×10^6^/ml of fresh CLL cells were treated with 20μg/ml of chlorambucil, cell death was determined by flow cytometry after 24 hours. **A.** and **B.** Correlation between chlorambucil-induced cell death and constitutive p-STAT3 (*n* = 15) or p-RelA (*n* = 15) expression. **C.** Correlation between spontaneous apoptosis and chlorambucil-induced cell death was analyzed in 38 CLL cases. **D.** Schematic illustration of the mechanisms by which STAT3/RelA co-regulate Bcl-xL and Mcl-1 expression, IL-6 production, and the chemoresistance.

We next determined whether the constitutive activity of STAT3 and NF-κB plays an important role in the resistance of CLL cells to chemotherapy. CLL cells were treated with chlorambucil (CBL) [24, 46] or fludarabine (FLU) [24, 47] for 24 hours. CBL or FLU-induced cell death were inversely correlated with both STAT3 and NF-κB activation (*p* < 0.001, Figure 6A, 6B and Supplementary Figure 3A, 3B). Importantly, the sensitivities of CLL cells to chemotherapy and spontaneous cell death were positively and significantly correlated (Figure 6C). Our results propose (Figure 6D) that, both constitutively activated STAT3 and NF-κB in CLL cells increased production of autocrine IL-6 and expression of Bcl-xL and Mcl-1. The CLL cells with higher p-STAT3 and p-RelA activities have better *in vitro* survival and higher chemoresistance. In turn, higher levels of IL-6 reflect greater constitutive activities of STAT3 and NF-κB.

### Spontaneous cell death reflects *in vivo* chemo-responsiveness and clinical outcome

**Figure 7:**
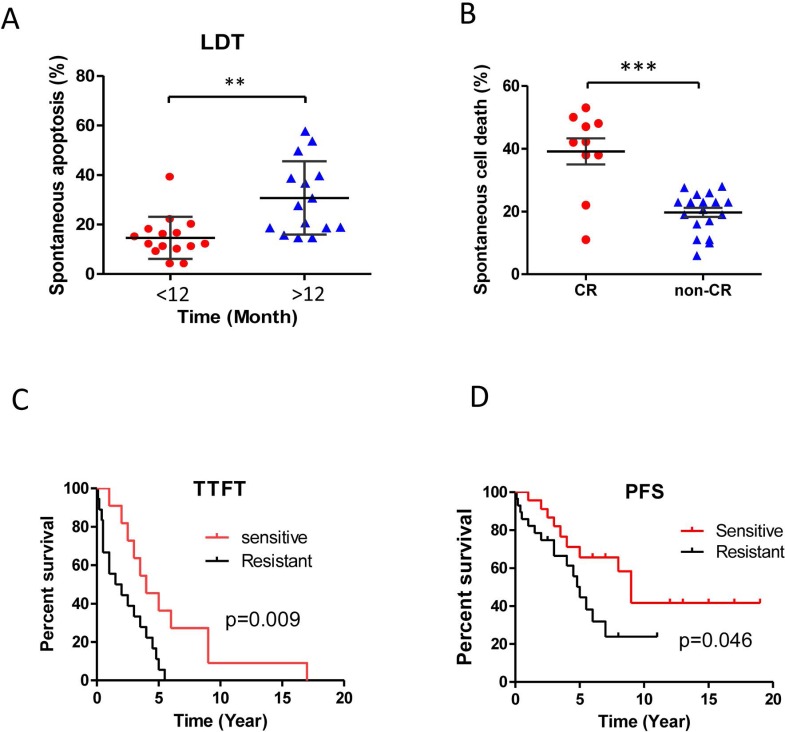
Spontaneous apoptosis and clinical prognosis Differential sensitivities to spontaneous apoptosis were analyzed. **A.** lymphocyte doubling time (LDT) more than 12 months and less than 12 months CLL cases; **B.** CLL cells from patients achieving a complete remission (CR, *p* < 0.01, *n* = 10) or non-CR (i.e., a partial response, stable disease and progressive disease; *n* = 19, *p* < 0.001). **C.** The significant difference between the CLL cells that were sensitive (i.e., more than average, *n* = 11) and those were resistant (less than average, *n* = 18) to spontaneous cell death in the time to first treatment (TTFT). **D.** The significant difference between the CLL cells that were sensitive (i.e., more than average, *n* = 23) and those that were resistant (less than average, *n* = 28) to spontaneous cell death in the progression free survival (PFS).

One of most intriguing features of CLL is its clinical heterogeneity with some patients progressing rapidly with early death but others exhibit a more stable non-progressive disease lasting many years. Thus, it is more important than ever to develop sensitive stratification parameters to identify patients with poor prognosis [48]. Lymphocyte doubling time (LDT), calculated by determining the number of months the absolute lymphocyte counts to be doubled, is widely used as measure of disease aggressiveness [48]. We assessed the correlation of LDT with *in vitro* spontaneous apoptosis and *in vivo* chemosensitivity. Patients were divided into two groups based on LDT longer than 12 months *versus* less than 12 months (Figure 7A); and their response to treatment: achieving a complete response (CR) *versus* not achieving a CR (i.e., a partial response, stable disease and progressive disease) (Figure 7B). CLL cases with a longer LDT (> 12 months) were more sensitive to spontaneous apoptosis (31%±14.8) *versus* the cases with a shorter LDT ( < 12 months; 15%±8.5, *p* < 0.01, Figure 7A) and spontaneous cell death in the CR group was significantly higher than those in non-CR group (Figure 7B, mean 39%±13 *versus* 19.7%±6.3 *p* < 0.001).

To further explore the correlation between spontaneous apoptosis and clinical prognosis, we analyzed the time to first treatment (TTFT) after diagnosis in the sensitive (more than average) and the resistant groups (less than average) to spontaneous apoptosis. The median of TTFT in the sensitive group was 4 years *versus* 1 year in the resistant group, while those cases with cells showing greater spontaneous apoptosis had longer TTFT (*p* < 0.05, Figure 7C). Furthermore, the median progression free survival (PFS) was significantly longer in patients with greater *in vitro* spontaneous apoptosis *versus* those cells were more resistant to spontaneous apoptosis (6 *vs* 3 years, *p* = 0.046, Figure 7D). These results indicate that the degrees of *in vitro* spontaneous apoptosis of CLL cells are not only associated with the *in vivo* speed of CLL cell accumulation and chemo-responsiveness but also associated with clinical progression of this disease.

## DISCUSSION

In this study, we demonstrate, for the first time, that the sensitivities of CLL cells to *in vitro* spontaneous and drug-induced apoptosis are associated with the constitutive activities of both STAT3 and NF-κB. Activation of these transcription factors up-regulates autocrine IL-6 production, and expression of anti-apoptotic proteins Mcl-1 and Bcl-xL, leading to resistance of CLL cells to *in vitro* spontaneous apoptosis and chemotherapy. Importantly, the resistance of CLL cells to spontaneous apoptosis reflects poor clinical outcome and disease progression.

CLL cells are long-lived and accumulate *in vivo* but undergo spontaneous cell death when they are cultured *in vitro* [3, 6, 49, 50], which is attributable to a loss of microenvironmental survival signals [34]. We found that *in vitro* spontaneous and drug-induced apoptosis of CLL cells is highly variable among different cases, suggesting that intrinsic cell mechanisms primarily dictate cellular fate in this setting. The sensitivity of CLL cells to spontaneous apoptosis has also been proposed to be associated with homotypic interaction between CLL cells [50], IgVH mutation status [6], or CD160 expression on CLL B-cells [4]. However, the underlying mechanisms and the association between spontaneous and drug-induced apoptosis in CLL cells are still unclear.

We aimed to study the relationship between the activity of STAT3/NF-κB and *in vitro* spontaneous apoptosis, and the underlying mechanisms. Transcriptional factors, STAT3 and NF-κB, play crucial roles in carcinogenesis, cancer cell proliferation and survival in many types of cancers [31, 33, 51, 52]. The activation and the interaction between STAT3 and NF-κB play a key role in controlling dialog between malignant cell and its microenvironment [44]. Previous studies have demonstrated different modes of STAT3 and NF-κB interaction in the transcriptional control: p-STAT3 interacts with p-RelA to recruit the p300 HAT complex leading to RelA acetylation that mediates STAT3/NF-κB dependent gene transcription [30, 44]. STAT3 interacts with p50/NF-κB and/or RelA together they induce gene transcription through binding to composite sites [44]. Using STRING database, it was predicated that STAT3 and NF-κB interact physically and functionally, suggesting they bind to the same or different promoters and activate gene expression. In CLL cells, we found that there is marked heterogeneity in constitutively activated STAT3 and RelA. The activation of STAT3 and RelA are significantly correlated with each other, which reflects their *in vivo* levels of activation at the time of phlebotomy. STAT3 and NF-κB (RelA) both control Bcl-xL and Mcl-1 expression, and IL-6 production. Inhibition or knockdown either p-STAT3 or p-RelA led to decrease in Bcl-xL/Mcl-1 expression and IL-6 production but an increase in spontaneous cell death. In view of this, the concentration of autocrine IL-6 reflects the levels of STAT3 and NF-κB activities. Therefore, a positive correlation between the resistance to spontaneous cell death and autocrine IL-6 production is unsurprising.

It is well known that Mcl-1 and Bcl-xL are critical mediators of malignant cell survival in CLL [4, 53, 54]. Both Bcl-xL and Mcl-1 are anti-apoptotic proteins, elevated expression of these proteins have been shown to prolong the survival of CLL cells exposed to a variety of apoptosis-inducing stimuli [55]. Furthermore, higher expression levels of Bcl-xL and Mcl-1 in CLL cells correlate with both poorer disease prognosis, and *in vivo* or *in vitro* chemo-resistance [53, 56, 57]. Our experiments demonstrated that STAT3 and NF-κB co-regulate Bcl-xL and Mcl-1 expression. Inhibition of either constitutive activation of STAT3 or NF-κB results in reduced both Bcl-xL and Mcl-1 expression but not Bcl-2. This finding explains the mechanism by which STAT3 and NF-κB prevent CLL cells from *in vitro* spontaneous apoptosis *via* sustaining the levels of Bcl-xL and Mcl-1. Similarly, the chemo-sensitization regulated by STAT3 and NF-κB is also dependent on Bcl-xL and Mcl-1 expression.

CLL is an accumulation disease that caused by failed *in vivo* spontaneous apoptosis or aberrant survival signal. LDT reflects *in vivo* CLL cell accumulation or disease progression [58]. The patients with LDT longer than 12 month showed more sensitive to spontaneous cell death. In contrast, the patients with LDT less than 12 month exhibited more resistance to spontaneous cell death. The clinical outcomes of CLL are significantly different in individual patients: some patients will live for decades and never require treatment, while others having aggressive disease require treatment at initial presentation [59]. The patients with sensitive CLL cells to spontaneous apoptosis had longer time to first treatment compare with the resistant individuals (median was 4 year *versus* 1 year). Therefore, the sensitivity of CLL cells to *in vitro* spontaneous apoptosis might predict the disease progression for patients with CLL.

The notion that both STAT3 and NF-κB support cell survival not only has been proved by spontaneous cell death but also testified by *in vitro* chemotherapy-induced cell death. CLL cells with higher activities of STAT3 or NF-κB manifested more resistant to drug-induced apoptosis; moreover the chemo-sensitivity was positively correlated with their sensitivity to spontaneous apoptosis. To evaluate *in vivo* response to chemotherapy in CLL cells, we found that most of CLL patients with complete response to the treatment are more sensitive to spontaneous apoptosis compared with the non-CR cells. In addition, the patients being more sensitive to spontaneous apoptosis have better progression free survival than the patients with resistance to spontaneous apoptosis group.

In summary, we demonstrated that constitutive activation of STAT3 and NF-κB plays important roles for *in vivo and in vitro* survival in CLL cells. The sensitivity of CLL cells to *in vitro* spontaneous apoptosis and autocrine IL-6 production reflects the status of constitutive activities of STAT3/NF-κB, chemo-responsiveness and clinical outcome of patients with CLL. We therefore propose that the sensitivity to spontaneous apoptosis could be used as a surrogate marker to predict chemo-responsiveness for CLL patients.

## MATERIALS AND METHODS

### Patients, cell separation, cell culture and treatment

The protocol was approval by the National Research Ethics Service, East London, and the City Health Authority Local Research Ethics Committee for *in vitro* studies on blood samples from CLL patients. Fifty-one patients were enrolled in this study and the cases either had never been treated or had not received chemotherapy or steroids for over 6 months (Supplementary Table). Peripheral blood was obtained after written informed consent [4]. Peripheral blood mononuclear cells (PBMC) were isolated by density-gradient centrifugation over Ficoll-Paque (GE Healthcare). Freshly isolated CLL cells were suspended in complete RPMI-1640 medium containing 10% fetal calf serum, and 2 mM L-glutamine and cultured at 37°C in a humidified incubator with 5% CO_2_.

### Reagents

The reagents used were as followings: anti-phosphorylated RelA, RelA and STAT3 were from Cell Signaling Technology; anti-Bcl-2 (100), anti-Bax (2D2), anti-Bcl-xL (s-18), and anti-Mcl-1 antibodies were purchased from Santa Cruz Biotechnology; anti-β-actin antibody was from Sigma-Aldrich; anti-p-STAT3 (phospho-STAT3-Ser-727, or p-STAT3^S727^) antibody and the Annexin V kit were purchased from BD Pharmingen; STAT3 inhibitors Stattic and 5-15 DPP were purchased from Sigma-Aldrich; RelA inhibitor *caffeic acid phenethylester* (CAPE) was from Alexis-Enzo Life Sciences and JSH-23 was from Calbiochem.

### Measurement of cytokines

Fresh CLL cells were isolated immediately after phlebotomy. The plasma was stored at −80°C. In all samples, the dual CD19+/CD5+ B-CLL cells were confirmed to represent more than 95% of the PBMCs. CLL B-cells in 5×10^6^/ml were incubated for 24 hours and the supernatants were stored at −80°C until were assayed. Cytokine analysis was performed using the human CBA Flex kit (BD Biosciences) for simultaneous measurement of IL-2, IL-4, IL-6, IL-10, TNF-α and VEGF, according to the manufacturer's instructions. Analysis was performed using a FACSCanto flow cytometer and FACSDiva software (BD Biosciences). The mean fluorescence intensity (MFI) was calculated using the CBA software (BD Biosciences) according to the standard curves and cytokine concentrations (picograms per milliliter, pg/ml). For IL-6 determination by ELISA, CLL cells were used immediately post-Ficoll (if CD5+/CD19+ cells were more than 95% of the PBMCs) or after magnetic-bead purification of PBMC (CD19+ CLL cells great than 95%), incubated for 24 hours (5×10^6^/ml) and IL-6 concentrations in the plasma or culture supernatants were measured according to the manufacturer's instructions (R&D Systems).

### Western blotting

Proteins were extracted with CelLytic^TM^ M cell Lysis Reagent (Sigma) supplied with protease inhibitor and phosphatase inhibitor cocktails (Sigma). Protein concentration was determined by the Bradford method, using the Bio-Rad Bradford reagent. Proteins were mixed with NuPAGE LDS Sample Buffer (Invitrogen) and boiled for 5 min. Proteins were then subjected to 4-20% NuPAGE gels (Invitrogen) and transferred onto PVDF membrane (Sigma) at 20 V for 1 hour by semi-dry transfer. PVDF membrane was blocked with 5% non-fat milk in TBST for 1 hour and then incubated with primary antibodies overnight at 4°C against the indicated targets. Bound antibodies were detected using appropriate HRP-conjugated secondary antibodies (Santa Cruz), visualized by GeneSnap (SynGene, Cambridge, UK) after adding ECL plus (GE Healthcare Life Science) [60, 61].

### Transfection of siRNA

Human STAT3, RelA, Mcl-1, Bcl-xL and control siRNA (Santa Cruz) were used to knockdown gene expression. Fresh primary CLL cells (5 × 10^7^/ml) were suspended in 100μl of Human B Cell Nucleofector (R) reagent (Lonza) and 2μg of siRNA was added into the mixture. Transfection was performed using Nucleofector^TM^ II apparatus with the program U-015 (Lonza). Transfection efficiency and protein expression were determined after transfection for 18 hours by flow cytometry or Western Blotting [60]. The cell death was determined by flow cytometry after transfection with PMAX-GFP plasmid for 24 hours.

### Determination of *in vitro* apoptosis

Apoptosis was determined using the Annexin V-FITC kit [49]. Briefly, fresh CLL cells were incubated with complete RPMI-1640 medium or treated by indicated cytokines or reagents. The percentages of Annexin V-FITC and propidium iodide (PI) positive cells were measured by flow cytometry (FACSCanto I). Annexin V-FITC and PI double-positive cells were defined as dead cells.

### Statistical analysis

Results are shown as mean ± SD or SEM from at least 4 individual cases. For statistical comparison between groups, the two-tailed paired or unpaired *t* test was used. GelScan V5.1 software was used to quantify protein expression on Western blots. The Pearson's Correlation method was used to analyze the difference between two variables Graphpad Prism software. All *P*-values less than 0.05 were considered statistically significant. *, ** and *** indicate *P*-value < 0.05, 0.01, and 0.001, respectively [62].

## SUPPLEMENTARY MATERIAL FIGURES AND TABLES


