# Supplementary material for: STAT3 and NF-κB cooperatively control in vitro spontaneous apoptosis and poor chemo-responsiveness in patients with chronic lymphocytic leukemia
Source: Oncotarget. 2016 Apr 9;7(22):32031–45. doi: 10.18632/oncotarget.8672 (PMC5077994; doi:10.18632/oncotarget.8672)
Supplement: Supplementary file 1 [file oncotarget-07-32031-s001.pdf]

STAT3 and NF-κB cooperatively control *in vitro* spontaneous apoptosis and poor chemo-responsiveness in patients with chronic lymphocytic leukemia

Supplementary Material

Suppl. Table: Study population of CLL clinical and laboratory characteristics.

		**No (%)**
**Total No of Subject***		51
**Sex**		
Male		29 (57)
Female		22(43)
**Age (Y)**		
Median		58
Range		38-83
**Lymphocytes (10^9/L)**		
Median		83.6
Range		12.1-350
**Binet stage**		
A		34(67)
B		9(18)
C		8(15)
**CD38 status**		
CD38+		16 (34)
CD38-		31 (65)
**Zap70 status**		
Zap70+		10 (38)
Zap70-		16 (61)
**Cytogenetic analysis**		
17p-/11q-		20(42)
Non 17p-/11q-		28 (58)
**Treatment status**		
Treated		29 (57)
Untreated		22 (43)
**Chemotherapy drug in used**		
**CR cases**	**10 (34)**	
FCR		5 (17)
FDR		3 (10)
Campath		1 (3)
FC		1 (3)
**Non CR cases**	**19 (66)**	
FCR		6 (21)
FDR		6 (21)
CBL		4 (14)

Campath	2 (7)
FC	1 (3)
**Follow up since diagnosis (Y)**	4-21

*Clinical data were not available for some patients: missing Zap70 expression (25/51), CD38 expression (3/51), cytogenetic analysis (3/51).

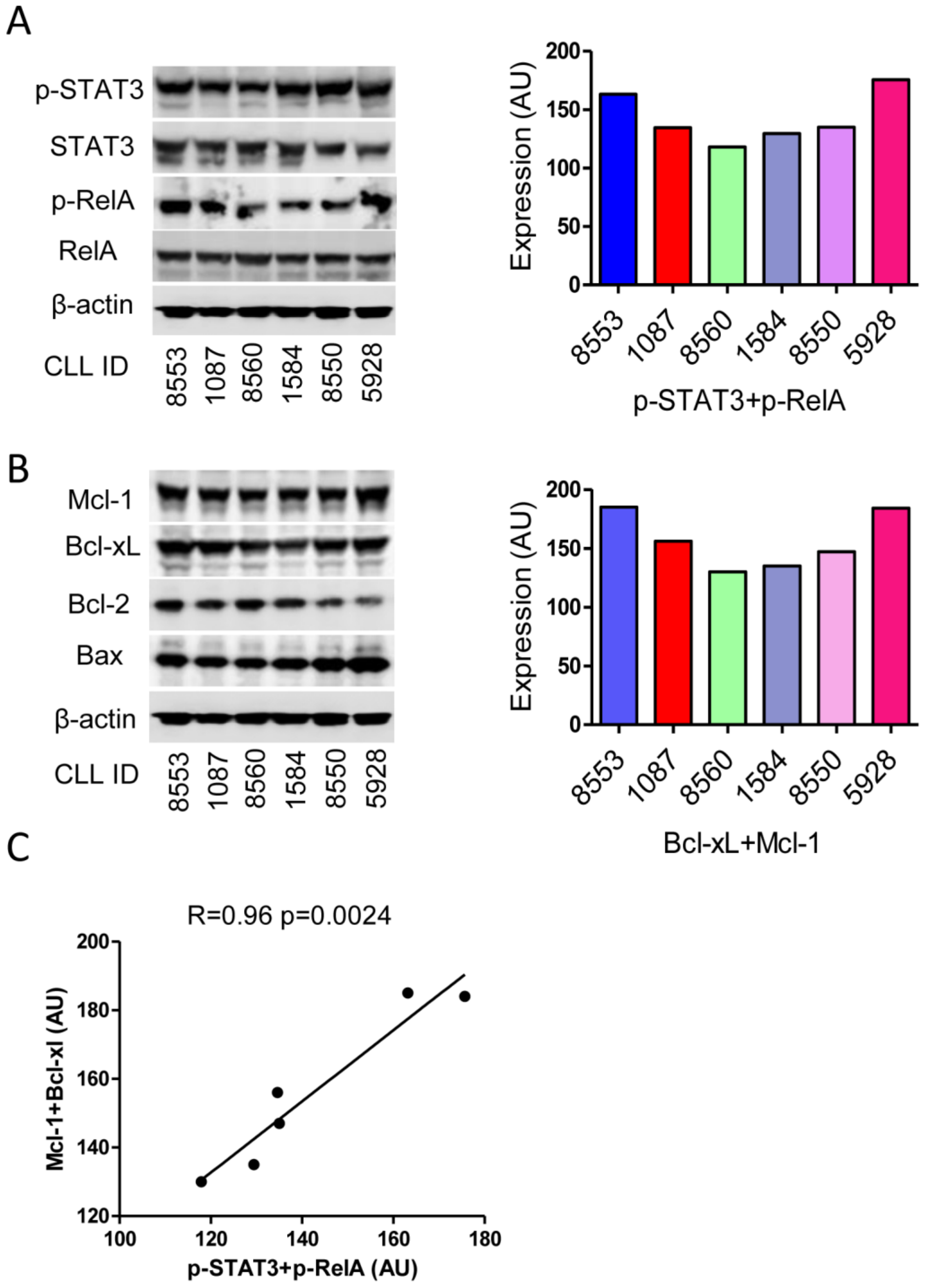

Supplementary Figure 1. Correlation of expression between Mcl-1+Bcl-xL and p-STAT3+p-RelA. Expression of p-STAT3 and p-RelA (A), Mcl-1 and Bcl-xL (B) in 6 freshly isolated CLL samples detected by Western blotting. (C) Correlation between Mcl-1+Bcl-xL and p-STAT3+p-RelA ($p < 0.01$).

A B

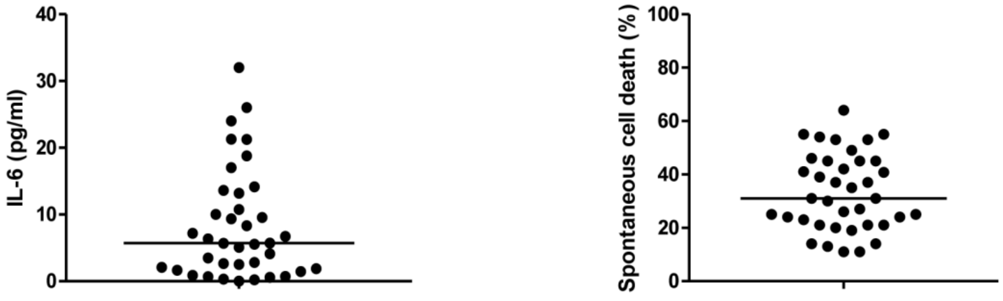

Supplementary Figure 2. Autocrine IL-6 production and spontaneous apoptosis. 38 freshly isolated CLL cells were incubated in culture medium, after 24 hours incubation, expression of IL-6 was measured by ELISA (A) and spontaneous apoptosis was detected by Flow Cytometry after 48 hours incubation (B).

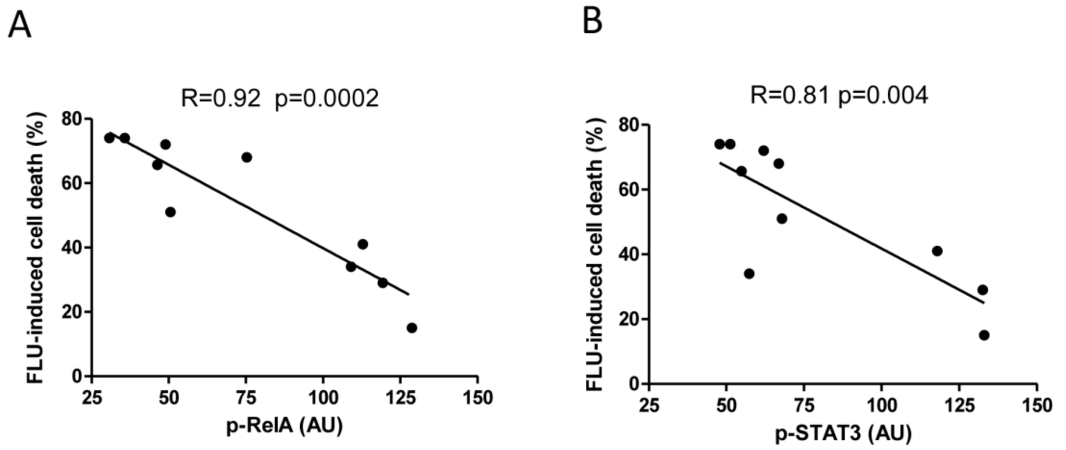

Supplementary Figure 3. Correlation between FLU-induced cell death with p-STAT3 or p-RelA. 10 fresh CLL samples were incubated with 20 µg/ml of Fludarabine for 24 hours. Correlation between Fludarabine-induced cell death with p-RelA expression $p<0.001$ (A); and with p-STAT3 expression $p<0.01$ (B).
